# SOFT ROBOTIC GLOVES VERSUS MIRROR THERAPY: A LONG-TERM COMPARATIVE STUDY ON HAND FUNCTION AND MOTOR RECOVERY IN POST-STROKE REHABILITATION

**DOI:** 10.2340/jrm.v57.43482

**Published:** 2025-08-28

**Authors:** Osama R. ABDELRAOUF, Mohamed A. ABDEL GHAFAR, Mariam E. MOHAMED, Zizi M. IBRAHIM, Eman M. HARRAZ, Mohamed K. SEYAM, Gihan Samir MOUSA, Rafik E. RADWAN, Amira E. EL-BAGALATY

**Affiliations:** 1 Physical Therapy Program, Batterjee Medical College, Jeddah, Saudi Arabia; 2 Department of Physical Therapy for Cardiovascular/Respiratory Disorder and Geriatrics, Faculty of Physical Therapy, Cairo University, Giza, Egypt; 3 Department of Rehabilitation Sciences, College of Health and Rehabilitation Sciences, Princess Nourah bint Abdulrahman University, P.O. Box 84428, Riyadh 11671, Saudi Arabia; 4 Department of Physical Medicine, Rheumatology and Rehabilitation, Faculty of Medicine, Mansoura University, Dakahlia, Egypt; 5 Department of Physical Therapy and Health Rehabilitation, College of Applied Medical Sciences, Majmaah University, Majmaah, Saudi Arabia; 6 Department of Physical Therapy, Faculty of Applied Medical Sciences, Umm Al-Qura University, Mecca, Saudi Arabia; 7 Department of Biomechanics, Faculty of Physical Therapy, Cairo University, Giza, Egypt; 8 Department of Physical Therapy for Pediatric, Faculty of Physical Therapy, Cairo University, Giza, Egypt

**Keywords:** hand function, mirror therapy, motor recovery, neurorehabilitation, soft robotic glove, stroke rehabilitation

## Abstract

**Objective:**

**This study aimed to compare the long-term effects of soft robotic gloves (SRGs) and mirror therapy on hand function and motor recovery in post-stroke patients.**

**Methods:**

**A single-blinded, randomized controlled comparative study was conducted on 49 patients with chronic stroke assigned randomly to either the SRGs or mirror therapy group. Both groups underwent an 8-week intervention alongside conventional rehabilitation. The Box and Block Test (BBT), hand grip strength, and Fugl-Meyer Assessment-Upper Extremity (FMA-UE), were assessed at baseline, post-intervention, and 6-month follow-up.**

**Results:**

**Post-intervention, the SRGs group demonstrated significantly greater improvements in all outcome measures compared with the mirror therapy group (*p* = 0.004, 0.011, and 0.021, respectively). These improvements were sustained at follow-up (*p* < 0.001, < 0.001, and 0.003, respectively). Within-group comparisons showed significant post-intervention improvements in both groups; however, the mirror therapy group exhibited no significant changes between post-intervention and follow-up (*p* = 0.197, 0.125, and 0.317, respectively), whereas the SRGs group maintained significant gains (*p* = 0.003, 0.012, and 0.005, respectively).**

**Conclusion:**

**Findings suggest that SRGs provide superior improvements in hand function and motor recovery compared with mirror therapy in post-stroke rehabilitation. The long-term benefits highlight the potential of SRGs as an effective intervention for promoting functional independence in stroke survivors.**

## LAY ABSTRACT

Post-stroke hand function impairment significantly affects activities of daily living, necessitating effective rehabilitation strategies. Mirror therapy (MT) and soft robotic gloves (SRGs) have emerged as promising interventions. To help stroke survivors regain hand function, we compared 2 rehabilitation methods. Our results showed that patients using the SRGs improved more than those using MT. These improvements lasted even after 6 months, while the MT group showed little long-term progress. This suggests that the robotic gloves could be a more effective way to support hand recovery, helping stroke survivors regain independence in daily life. Our findings highlight the potential of this technology to improve rehabilitation outcomes and quality of life for stroke patients.

Stroke remains a major global health concern, ranking as the second leading cause of death and the third leading cause of disability, according to the World Stroke Organization (1). A significant proportion of stroke survivors, approximately 60%, experience chronic dysfunction, with 60–80% suffering from upper-limb motor impairments (2). Unfortunately, only 5–20% of these patients fully recover their upper-limb functionality, and the degree of recovery varies depending on multiple factors, such as stroke severity, rehabilitation intensity, and neuroplastic potential (3).

The loss of hand function poses significant challenges, making it difficult for individuals to grip, hold, and manipulate objects effectively. This impairment significantly impacts their ability to perform activities of daily living (ADLs), ultimately reducing independence, limiting social participation, and diminishing overall quality of life (4).

Rehabilitation strategies for post-stroke hand function recovery are primarily based on activity-dependent therapy, which leverages principles of neuroplasticity. Intensive, interactive, high-repetition, and task-specific training is an effective approach to regaining hand function after a stroke (5). However, despite these advancements, rehabilitation programmes often struggle to provide the level of intensity needed for optimal recovery to all patients. This limitation highlights the need for novel, accessible, and scalable rehabilitation interventions (6).

Mirror therapy (MT) activates the mirror-neuron system, allowing the brain to perceive movement in paralyzed limbs, which in turn activates inactive motor pathways following a stroke. The evidence supporting mirror therapy has strengthened over the past 2 years, as a 2025 meta-analysis of 18 randomized trials involving 633 participants demonstrated significant improvements in upper-limb motor scores and dexterity when patients practised more than 5 times a week during the first month (7). The combination of mirror feedback with functional electrical stimulation (FES) in Korean clinical settings resulted in improved Fugl-Meyer scores and increased wrist range in patients with chronic conditions (8). Meanwhile, Taiwanese researchers found that using mirror cues with neuromuscular stimulation improved both gait speed and balance (9).

The use of technology has enhanced the maximum potential for recovery. The significant effect size of virtual-reality (VR) mirror systems on upper-extremity recovery persists for 3 months after training completion, indicating that the benefits extend beyond the period of mirror usage (10). The programmes provide evidence of small but significant reductions in spasticity that occur after the initial sub-acute recovery phase (11). Research evidence supports that mirror therapy yields its best results through intensive delivery and when combined with other multi-sensory protocols, creating a low-cost approach to achieving lasting functional improvements for stroke survivors.

Simultaneously, technological advancements have led to the development of soft robotic devices, particularly soft robotic gloves (SRGs), which aim to support functional hand recovery. Recent high-quality trials have strengthened the evidence base for soft-robotic gloves (SRGs) in stroke care. A single-centre randomized controlled trial (RCT) of an EMG-triggered, patient-specific glove (20 × 45-min sessions) involving 34 chronic survivors produced significantly larger gains than dose-matched conventional practice in distal FMA-UE, Box and Block, and active finger range of motion (ROM), with benefits still present 3 months later (12). Complementary data from a sub-acute, single-blind RCT (*n* = 40) show that 4 weeks of task-oriented therapy with a haptic, force-feedback glove (SEM™) results in greater improvements in FMA-Hand, and grip strength compared with therapist-assisted practice, without increasing spasticity or adverse events (13).

Taken together, these studies confirm that modern SRGs can provide intensive, sensorimotor-rich practice that translates into clinically meaningful recovery of grasp strength, dexterity, and proximal control across both chronic and sub-acute phases. At the synthesis level, a 2025 meta-analysis of 15 RCTs (*n* = 574 participants) reported a large effect of robot-assisted task-oriented training on FMA-UE and a moderate effect on independence. Subgroup analysis indicated that soft-glove studies were the primary drivers of hand dexterity and ADL gains (14). However, large multicentre trials with a follow-up period of at least 6 months and cost-effective endpoints remain a priority.

While both MT and SRGs have demonstrated potential in post-stroke rehabilitation, most existing research has focused on short-term outcomes, typically within a 4- to 12-week timeframe. This leaves a critical gap in understanding their long-term effects on motor recovery. A recent meta-analysis found that rehabilitation incorporating SRGs resulted in significant improvements in hand functions both immediately after the intervention and during follow-up assessments (7). In contrast, studies on MT have shown mixed results regarding long-term benefits. Some research has reported sustained improvements in motor function at 6-month follow-ups, while others have found that gains diminish over time (8, 9).

Given the severity of upper-limb impairment in hemiplegic patients and the prolonged rehabilitation process required for meaningful recovery, assessing the long-term efficacy of these interventions is essential. Determining whether MT and SRGs provide lasting functional improvements or if continued maintenance programmes are necessary could significantly influence future rehabilitation strategies. To address this gap in the literature, this randomized controlled comparative study aimed to investigate this PICOT question: “In adults with chronic stroke (P), does an 8-week programme using soft robotic gloves (I) compared with mirror therapy (C) lead to greater and sustained improvements in hand function, grip strength, and upper-extremity motor recovery (O) over a six-month follow-up period (T)?”

## METHODS

### Study design

This single (assessor)-blinded, randomized controlled comparative study evaluated the effectiveness of a soft robotic glove vs MT in improving hand function and motor recovery in patients undergoing post-stroke rehabilitation. The study was conducted across 3 local long-term inpatient rehabilitation centres over a 15-month period, from July 2023 to December 2024, in accordance with the CONSORT guidelines.

The Ethical Committee of Batterjee Medical College (RES-2024-0217) reviewed and approved the study procedures in accordance with the latest version of the Declaration of Helsinki. Additionally, the study was registered on ClinicalTrials.gov on 1 June 2023, under the ID NCT05898971.

### Participants

Forty-nine post-stroke patients (29 male and 20 female) participated in this study. A confirmed diagnosis of stroke, verified through medical imaging (CT or MRI), indicates the presence of a cerebrovascular event. This criterion ensures that all participants have a medically verified stroke diagnosis, maintaining a consistent and well-defined study population. Initially, 55 residents were screened to determine their eligibility based on the study’s inclusion criteria. The screening process was conducted after obtaining formal approval from the management of these rehabilitation centres, ensuring alignment with the study’s objectives and procedures.

The inclusion criteria for the study comprised individuals aged 40–80 years (10) with first-ever unilateral chronic stroke, defined as more than 6 months post-stroke onset (11), and moderate to severe upper limb paresis, as objectively assessed by the Fugl-Meyer Assessment-Upper Extremity (FMA-UE) scale, with scores ranging from 15 to 45 out of 66, indicating moderate impairment (12), hypertonia grade 1, 1+, or 2 on the modified Ashworth scale (13). Also, the participants were required to be able to sit for at least 30 min, have stable medical status, and demonstrate the ability to comprehend and consistently follow verbal and written instructions (14).

The exclusion criteria include the following: significant cognitive impairment that hinders participation, as assessed by the Mini-Mental State Examination with a score < 24 (15); the presence of concurrent neuromuscular conditions that may independently impact upper limb function; severe shoulder subluxation; and any contraindications to MT or robotic therapy, such as physical limitations, allergies, or medical conditions that could pose a risk to the participant’s safety.

The study employed a stratified random sampling technique to assign participants into 2 intervention groups: 1 receiving training with an SRGs and the other undergoing mirror therapy. Additionally, both groups participated in conventional physical therapy sessions as part of their rehabilitation programme. Stratification was applied to control for specific variables, particularly sex and the affected side, ensuring a more balanced and representative sample.

To maintain allocation concealment, participant assignments were placed in opaque, sealed, and consecutively numbered envelopes, thereby minimizing the risk of selection bias. These envelopes were securely stored and only opened during group allocation. An independent third party, uninvolved in participant recruitment or intervention, was responsible for opening the envelopes, further securing the integrity of the randomization process.

**Fig. 1:**
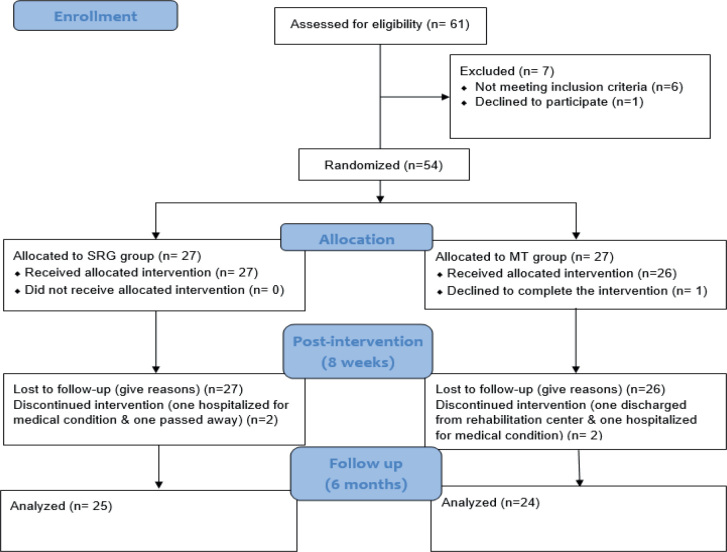
Flowchart of study.

Following a thorough description of the study’s procedures, risks, and benefits, participants provided their signed informed consent, ensuring their voluntary and informed participation. The study flowchart is presented in Fig. 1. It tracks the study population from screening to final analysis in full CONSORT format:

*Screening and randomization:* 61 individuals were assessed for eligibility; 7 were excluded (6 did not meet the inclusion criteria, 1 declined), leaving 54 participants who were randomized (27 to SRG, 27 to MT).

*Treatment allocation:* All 27 SRG participants and 26 of 27 MT participants started their assigned intervention (1 MT participant withdrew before the first session).

*Post-intervention (8 weeks): SRG:* Two participants were discontinued (1 hospitalized for an unrelated medical condition; 1 passed away).

*MT:* Two discontinued (1 discharged from the rehabilitation centre; 1 hospitalized for a medical condition).

*Six-month follow-up and analysis:* After accounting for these withdrawals, 25 SRG and 24 MT participants completed follow-up and were included in the primary and secondary analyses, yielding overall completion rates of 92% and 88%, respectively.

### Sample size calculation

The sample size was determined through power analysis using G*Power 3.1 (https://www.psychologie.hhu.de/arbeitsgruppen/allgemeine-psychologie-und-arbeitspsychologie/gpower), aiming for a medium effect size (Cohen’s *d* = 0.6) based on expected differences in the Box and Block Test (BBT) (16). With a significance level (α) of 0.05 and 80% statistical power, the analysis indicated that a minimum of 44 participants was required to detect meaningful differences between groups. To account for a potential 15% attrition rate, 50 participants were enrolled in the study. This ensured that the study maintained sufficient power to draw reliable conclusions, even in the case of participant dropout.

### Outcome measures

All outcome measures were evaluated at baseline (pre-intervention) and after 8 weeks of intervention with SRGs training or MT (post-intervention). Additional measurements were taken 6 months after completing mirror and robotic glove therapy (long-term follow-up). To minimize assessment bias, the evaluators were blinded to the participants’ group assignments, ensuring that the measurements remained objective and unbiased throughout the study.

*Primary outcome: gross manual dexterity as measured by the BBT.* BBT is a widely recognized assessment tool for evaluating unilateral gross manual dexterity, particularly in individuals with neurological conditions such as stroke (17). The BBT has strong psychometric properties, demonstrating good construct validity through its correlation with other measures of manual dexterity and functional ability (18). It exhibits excellent test–retest reliability (ICC = 0.93–0.98) and high inter-rater reliability (ICC = 0.99–1.000), making it a highly reliable tool for assessing manual dexterity in stroke patients (19). While the BBT is well suited for individuals with mild to moderate hemiparesis, it may not be suitable for those with severe hemiparesis, as they may struggle to perform the task effectively. Additionally, the BBT is moderately sensitive to changes in manual dexterity post-stroke, making it a valuable measure for tracking rehabilitation progress over time (20).

*Secondary outcomes: hand grip strength as measured by the HandHeld Dynamometer (HHD).* Hand grip strength was measured by Baseline Hydraulic HHD (Product 12-0240, SN 04200579, Fabrication Enterprises Inc, USA). It features a dual-scale readout displaying isometric grip force from 0–90 kg (0–200 lb) and has a peak hold needle that retains the highest reading until reset.

Grip strength measurements using HHD are considered valid indicators of overall muscle strength and health status. They correlate well with other measures of upper limb function and are predictive of clinical outcomes (21). Studies have shown that grip strength measurements using dynamometers are highly reliable, with excellent test–retest reliability (ICC = 0.97–0.99) and minimal detectable differences (MDD = 2.73–4.68 kg) 26 (22).

*Motor recovery as measured by FMA-UE Scale.* The FMA-UE is a part of the broader Fugl-Meyer Assessment (FMA), which evaluates sensorimotor impairment following stroke. The FMA-UE has demonstrated excellent construct validity, correlating well with other measures of motor function and recovery post-stroke. It is responsive to changes in motor function over time, making it suitable for tracking progress in recovery (23). Both intra-rater and inter-rater reliability of the FMA-UE are excellent, with intra-class correlation coefficients (ICC) typically above 0.90. This indicates high consistency in scoring across different raters and assessments (24).

FMA-UE is highly recommended for assessing upper limb motor function in chronic post-stroke patients, as it is sensitive to changes in motor function, making it useful for evaluating the effectiveness of interventions aimed at improving upper limb function in stroke patients. The minimal clinically important difference for the FMA-UE is reported to be 9–10 points for individuals poststroke, highlighting its responsiveness to clinically meaningful changes (25).

### Procedures

*Assessment procedures.* After collecting demographic data, participants underwent a comprehensive baseline assessment of manual dexterity, grip strength, and upper extremity motor recovery. Manual dexterity was evaluated using BBT, grip strength was measured with HHD, and upper limb motor recovery was assessed using the FMA-UE. To minimize the potential impact of fatigue on measurements, the order of the assessment tests was randomized for each participant.

The BBT consists of a wooden box divided into 2 compartments by a partition, a stopwatch, and 150 coloured wooden blocks, each measuring 1 inch (2.5 cm) in diameter. During the assessment, the participants were asked to sit at a table facing the examiner, with the box in front of them. All blocks were initially placed in a single compartment. The task required the participant to move as many blocks as possible from one compartment to the other within 60 s, ensuring that each block crosses over the partition to be counted. The final score represents the total number of blocks successfully transferred within the time limit (26).

For hand grip strength measurements, participants sat with their feet flat on the floor and their arms at a right angle, with their elbows beside their bodies. The dynamometer handle was adjusted to fit the participant’s hand, and they were instructed to squeeze as hard as possible, and the mean reading of 3 trials was recorded (27). The FMA-UE assesses motor function in the upper extremity, with a maximum score of 66 points. It assesses the ability to perform various movements and tasks, including shoulder flexion, elbow flexion, forearm supination, wrist flexion, and finger flexion and extension. Each item is scored on a 3-point ordinal scale: 0 (cannot perform), 1 (performs partially), and 2 (performs fully) (28).

*Intervention.* Both groups received a standardized conventional physical therapy programme for 45 min, 5 days a week, which included a combination of stretching, strengthening, endurance training, balance and coordination exercises, range of motion activities, and overground walking practice. These interventions were designed to enhance overall motor function, improve mobility, and support functional recovery (18). Conventional physical therapy was followed immediately by 30 min of the assigned intervention: mirror therapy or soft-robotic-glove training. Placing conventional therapy first both primed the limb for fine-motor practice (reducing spasticity and boosting cortical excitability) and guaranteed that all participants received the same dose of usual care before the experimental component.

*MT group.* A mirror measuring 33 cm × 38.6 cm (North Coast Medical Inc, Morgan Hill, CA, USA) was used for training in the MT group. The MT session was conducted in a quiet, distraction-free environment. The mirror was positioned at the patient’s midline, ensuring that the reflection of the unaffected limb appeared as the affected limb. Its size provided a clear and complete reflection of the moving limb. The patient sat comfortably in front of the mirror with one arm on either side. The affected limb was positioned behind the mirror, out of sight. The MT training session was structured as follows.

**Fig. 2:**
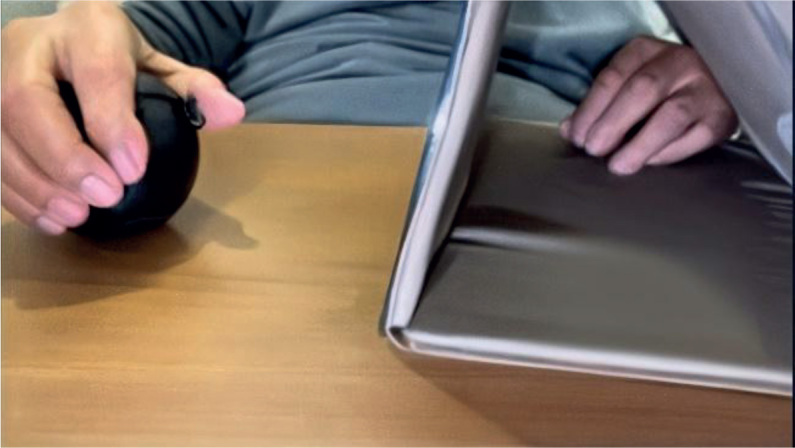
Mirror therapy (grabbing and rolling a ball).

Warm-up (5 minutes): The patients performed gentle range-of-motion exercises with their unaffected right arm, focusing on its reflection in the mirror. These exercises included finger flexion and extension, thumb–finger opposition, wrist flexion and extension, wrist radial and ulnar deviation, forearm supination and pronation, and elbow flexion, with 10 repetitions for each movement. Functional tasks (10 min): The patients performed functional tasks with the unaffected right arm while focusing on its reflection in the mirror. These tasks included grasping small objects (e.g., stress balls, blocks) and placing them in a container, reaching for targets at varying distances and heights, picking up and releasing objects of different sizes, and stacking blocks (Fig. 2). As the session progressed, more complex activities were introduced, such as writing or drawing simple shapes and manipulating therapy putty. Bilateral movements (10 min): The patient performed bimanual movements, with the unaffected right arm leading and the affected left arm attempting to follow. These tasks included range-of-motion exercises and functional activities, such as folding a towel and wiping a table. Cool-down (5 min): The session concluded with gentle range-of-motion exercises for both arms, integrated with controlled breathing to promote relaxation and reduce any tension accumulated during the training. The MT training protocol used in this study was adapted from the intervention procedures described in previous studies (29, 30). The exercise protocol was progressively advanced as the patient improved. This progression involved gradually increasing repetitions, adding resistance, and enhancing task complexity.

*SRGs group*. Soft Rehabilitation Gloves (Shanghai SiYi Intelligent Technology Co, Ltd, Shanghai, China) were used. High-repetition training is crucial for promoting neuroplasticity and facilitating motor recovery following a stroke. The SRGs facilitate this by providing support, enabling patients to perform more repetitions than they could independently (31). Active-assisted movement is a key principle, where the patient initiates the movement, and the robotic glove helps only as needed to complete the motion (32).

**Fig. 3:**
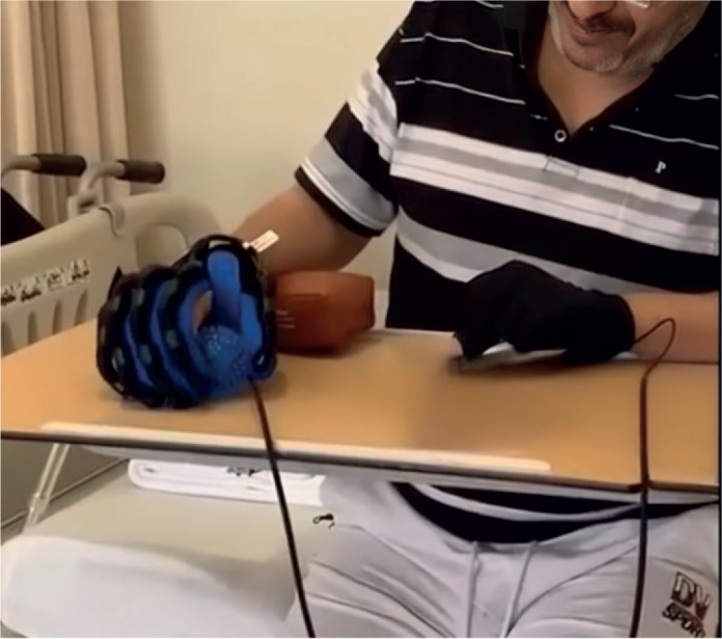
Robotic gloves (grabbing and rolling a ball).

The glove was adjusted to fit the patient’s hand properly, ensuring it neither restricted circulation nor caused discomfort. The therapist also ensured that the glove was properly aligned, with the actuators positioned correctly to assist the desired movements (33). The components of an SRGs therapy session were identical to those of the MT group, with a stronger emphasis on sagittal plane movements, where the glove provides the most support. The intensity of the exercises can be adjusted by modifying the assistance level of the SRGs. Initially, the glove provides high assistance to aid movement, which is gradually reduced as the patient’s strength and control improve, encouraging more active use of their muscles (Fig. 3).

In addition to conventional physical therapy, MT and SRGs were provided for 30 min, 5 times a week, over an 8-week period. After these 8 weeks, conventional physical therapy continued for an additional six months.

### Statistical analysis

Statistical analysis was carried out using IBM SPSS Statistics for Windows, version 25 (IBM Corp, Armonk, NY, USA). Before proceeding with the main analysis, the data were screened to ensure they met the assumptions of normality. This involved assessing normality using Levene’s test and the Shapiro–Wilk test, as well as evaluating skewness, kurtosis, and the presence of extreme values. Parametric tests were applied once the data were confirmed to adhere to normality assumptions.

To assess differences in mean values across all measured outcomes between the two groups, a repeated-measures multivariate analysis of variance (MANOVA) was performed. When statistically significant differences were identified, *post hoc* comparisons were conducted using the Bonferroni correction to control for multiple comparisons.

A significance level of 0.05 was set for all analyses. Treatment effects were reported as between-group differences. Additionally, standardized mean differences (Cohen’s *d*) were calculated, along with 95% confidence intervals, to quantify the magnitude of the treatment effect. Effect size values were interpreted as follows: 0.20 indicating a small effect, 0.50 a moderate effect, and 0.80 or greater a large effect (34).

## RESULTS

Baseline comparisons revealed non-significant differences in all tested demographic and clinical characteristics between the SRGs and MT groups, as presented in Table I.

**Table I:** Demographic and clinical characteristics of the SRGs and the MT groups

Item	SRGs group(*n* = 25)	MT group(*n* = 24)
Age, years, mean (SD)^a^	64.8 (11.4)	61.3 (13.6)
Stroke latency, months, mean (SD)^a^	8.0(0.8)	7.6 (1.2)
Gender, M/F, *n*^b^	15/10	14/10
Affected side, *n*^b^	18/4	16/8
Hypertonia, 1,1+/2^b^	13/12	14/10
Lesion type, ischaemic/haemorrhagic^b^	21/4	22/2

SRGs: soft robotic gloves; MT: mirror therapy; SD: standard deviation.

The repeated-measures MANOVA revealed significant Group × Time interaction effects for all outcome measures (Wilks’ λ = 0.62, *F*(6, 42) = 4.31, *p* = 0.002, η² = 0.38), indicating differential improvements between the SRGs and MT groups over time. *Post hoc* analyses with Bonferroni correction showed that the SRGs group exhibited significantly greater improvements in gross manual dexterity (BBT: *F*(2, 47) = 18.76, *p* < 0.001, η² = 0.44), grip strength (*F*(2, 47) = 12.53, *p* < 0.001, η² = 0.35), and upper-extremity motor recovery (FMA-UE: *F*(2, 47) = 9.84, *p* = 0.003, η² = 0.30) compared with the MT group. The interaction effects were driven by sustained gains in the SRGs group at both post-intervention and 6-month follow-up (*p* < 0.01 for all measures). In contrast, the MT group’s improvements plateaued after the intervention (*p* > 0.05 for follow-up vs post-intervention).

### Primary (between-group) analyses

*Gross manual dexterity (BBT – primary outcome).* At baseline, the 2 groups did not differ in Box and Block Test scores (*p* = 0.253; *d* = 0.25). After the 8-week intervention, the SRGs group demonstrated a significantly greater gain compared with the MT group (*p* = 0.004, MD = 5.34, 95% CI = 2.35 to 8.32, and *d* = 0.86). This advantage not only persisted but also increased at the 6-month follow-up (*p* < 0.001, MD = 8.8, 95% CI = 4.56 to 13.03, and *d* = 1.18), indicating a strong and lasting effect of the SRGs programme on gross manual dexterity.

### Secondary outcomes

*Hand-grip strength (HHD).* Baseline hand-grip strength did not differ between groups (*p* = 0.568, MD = 4.1, 95% CI = 0.94 to 7.25, and *d* = 0.19). The SRGs group outperformed the MT group post-intervention (*p* = 0.011; *d* = 0.76) and retained a superior grip at 6 months (*p* < 0.001, MD = 5.99, 95% CI = 2.67 to 9.3, and *d* = 1.03).

*Upper-limb motor recovery (FMA-UE).* No pre-intervention difference was observed on the Fugl-Meyer Upper Extremity scale (*p* = 0.246, MD = 6.28, 95% CI = 1.1 to 10.45, and *d* = 0.34). SRGs participants achieved higher FMA-UE scores than MT participants at 8 weeks (*p* = 0.021; *d* = 0.67) and at the 6-month follow-up (*p* = 0.003, MD = 7.23, 95% CI = 1.04 to 12.41, and *d* = 0.79).

### Secondary (within-group) analysis

*Gross manual dexterity – Box and Block Test (primary outcome)*

*SRGs group:* Dexterity rose sharply from baseline to the 8-week mark (*p* < 0.001, MD = 13.19, 95% CI = 11.51 to 17.86, and *d* +1.74) and continued to gain ground between post-test and the 6-month follow-up (*p* = 0.003, MD = 6.02, 95% CI = 2.05 to 9.98, and *d* = 0.86), indicating a sustained and progressive benefit.

*MT group:* Dexterity also improved from baseline to post-test (*p* = 0.002, MD = 5.75, 95% CI = 2.88 to 8.61, and *d* = 0.84) but then levelled off; no additional change was detected over the subsequent 6 months (*p* = 0.197, MD = 2.56, 95% CI = –0.78 to 5.9, and *d* = 0.47).

### Hand-grip strength – Hand-Held Dynamometer (secondary outcome)

*SRGs group:* Grip strength improved significantly after the intervention (*p* < 0.001, MD = 7.48, 95% CI = 4.77 to 10.18, and *d* = 1.57) and continued to rise through follow-up (*p* = 0.012, MD = 4.32, 95% CI = 0.66 to 7.97, and *d* = 0.83).

*MT group:* A modest but significant gain was seen at post-test (*p* = 0.038, MD = 3.02, 95% CI = 0.18 to 5.85, and d = 0.58), with values remaining stable thereafter (*p* = 0.125, MD = 2.43, 95% CI = –0.14 to 4.75, and *d* = 0.42.

### Upper-limb motor recovery – FMA-UE Scale (secondary outcome)

*SRGs group:* FMA-UE scores climbed from baseline to post-test (*p* < 0.001, MD = 13.12. 95% CI = 9.84 to 18.39, and *d* = 1.36) and showed an additional increment by 6 months (*p* = 0.005, MD = 5.57, 95% CI = 0.12 to 11.26, and *d* = 0.79).

*MT group:* Scores improved up to post-test (*p* = 0.029, MD = 5.54, 95% CI = 0.68 to 10.39, and *d* = 0.64) but did not change further during follow-up (*p* = 0.317, MD = 4.62, 95% CI = –1.08 to 10.32, and *d* = 0.46).

**Fig. 4:**
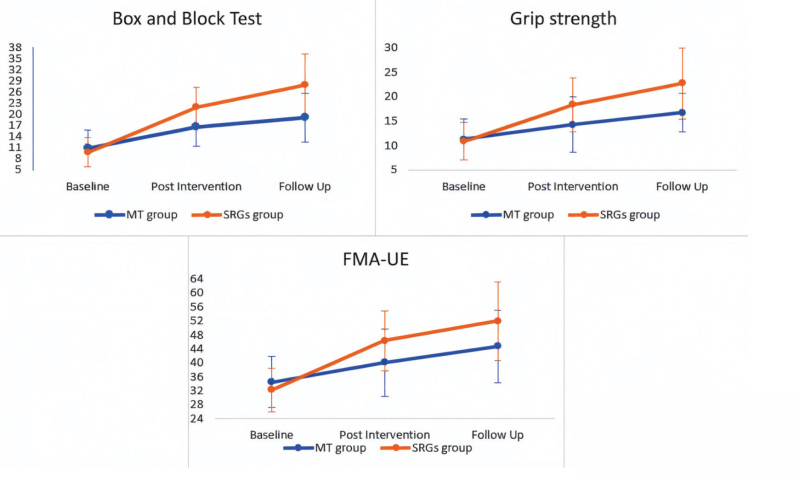
Pattern of change in outcome measures for both groups from baseline through post-intervention to follow-up assessments.

The results of the between-group and within-group comparisons are presented in Table II and Fig. 4.

**Table II:** Between-group and within-group comparisons for all outcome measures

Variables	Groups	BaselineMean (SD)	Post-interventionMean (SD)	Follow-upMean (SD)	Pre- vs post-intervention comparison		Post-intervention vs follow-up comparison	
					*P* value	Effect size	*P* value	Effect size
Box and Block Test	MT group	10.82 (4.93)	16.57 (5.14) 7.02	19.13 (6.53)	0.002*	0.84	0.197	0.47
	SRGs group	9.72 (3.96)	21.91 (5.36)	27. 93 (8.27)	< 0.001*	1.74	0.003*	0.86
	*p*-value	0.379	0.004*	< 0.001*				
	Effect size	0.25	0.85	1.18				
Grip strength	MT group	11.23 (4.25)	14.25 (5.62)	16.68 (3.92)		0.58	0.125	0.42
	SRGs group	10.87 (3.91)	18.35 (5.48)	22.67 (7.25)		1.57	0.012	0.83
	*p*-value	0.568	0.011*	< 0.001*				
	Effect size	0.18	0.75	1.02				
FMA-UEtotal	MT group	34.55 (7.36)	40.09 (9.58)	44.71 (10.45)		0.64	0.317	0.46
	SRGs group	32.25 (6.28)	46.37 (8.58)	51.94 (11.28)		1.36	0.005*	0.79
	*p*-value	0.246	0.021	0.003				
	Effect size	0.33	0.67	0.78				

SRGs: soft robotic gloves; MT: mirror therapy; FMA-UE: Fugl-Meyer Assessment-Upper Extremity; SD: standard deviation.

* *p*-value indicates a significant difference at alpha 0.05.

## DISCUSSION

In stroke-rehabilitation research, a follow-up conducted at least 3 months after therapy is generally considered long term because most neuro-motor improvements plateau by then, and the main question is whether the gains are maintained (35). Our study re-evaluates participants 6 months post-treatment, extending the assessment beyond this point to evaluate the durability of recovery during the crucial maintenance phase. This extended observation period clearly defines the current work as a long-term follow-up study.

The findings of this study suggest that SRGs offer superior benefits compared with MT in improving manual dexterity, grip strength, and UE motor recovery in post-stroke patients. Post-training evaluation demonstrated short-term training outcomes, while a 6-month follow-up assessment confirmed the consistent training effect. The results indicated that the SRGs group exhibited sustained improvements in all outcome measures at the 6-month follow-up. In contrast, the MT group did not maintain their initial gains over the same period. These findings suggest that integrating SRGs therapy with traditional physical therapy may facilitate long-term functional recovery in post-stroke hand rehabilitation.

The sustained efficacy of SRGs therapy may be attributed to its capacity to provide repetitive, task-specific training, which is essential for neuroplasticity and motor learning. By delivering consistent assistance and sensory feedback during hand movements, SRGs facilitate the reinforcement of neural pathways associated with motor function, leading to lasting improvements. In contrast, MT relies on visual feedback and mental imagery, which may not sufficiently engage the neural circuits required for enduring motor recovery, particularly in patients with severe impairments (32).

### Manual dexterity

Both interventions enhanced dexterity, but SRGs produced the larger and more durable gains. The SRG group demonstrated significant, clinically important improvements on dexterity tests, which were sustained at the 6-month follow-up. In a recent randomized controlled trial by Li et al. (36), the SRG group demonstrated significant and clinically meaningful improvements on standard dexterity measures such as the Action Research Arm Test (ARAT), by 6–8 points, and the Box and Block Test (BBT), by over 10 blocks, surpassing the minimal clinically significant difference. These improvements were sustained at the 6-month follow-up.

On the other hand, MT elicited early benefits at post-intervention that slowed down over the same period, confirming earlier reports by Yavuzer et al. (8) and Corning and Hildebrand (37) that the visual imagery demands of MT are less effective in the chronic phase. Moreover, a 2025 meta-analysis including 15 SRG trials confirmed a significant short-term effect on dexterity, with improvements retained for at least 12 weeks (38). Additionally, home-use investigations have supported these findings (39). In contrast, a parallel meta-analysis of 15 MT RCTs by Hsieh et al. revealed a moderate short-term effect that declined to a small but significant long-term effect (40). These different treatment trajectories underscore the importance of repetitive, sensorimotor-rich glove practice in consolidating fine-motor skills (41).

### Hand grip strength

Recent randomized controlled trials have demonstrated that SRGs, particularly those incorporating force feedback or electromyography (EMG)-triggered activation, significantly enhance grip strength over 3–6 weeks of training. For instance, the EMG-driven glove used by Shi et al. (42) produced significant improvements in both grip strength and active finger range of motion. These results are likely attributable to the ability of SRGs to deliver high-intensity, task-specific resistance training, with real-time feedback that actively engages the neuromuscular system during each grasp attempt. In contrast, MT’s effects on grip strength are minor and more variable. While some studies report mild to moderate short-term gains (≈0.5–1.0 kg) (8), others find no significant difference compared with control therapy (43).

The differences between our findings and those of previous studies on mirror therapy may stem from several factors, including variations in sample size, methodological differences in intervention protocols, and, importantly, differences in participant characteristics such as baseline spasticity and functional level. Patients with more severe impairment may be less able to benefit from visual feedback, which could explain the reduced effectiveness observed in some cases.

### Upper-extremity motor recovery

Distal practice translated into proximal recovery was observed in both groups as measured by the Fugl-Meyer Assessment for the Upper Extremity (FMA-UE). However, the magnitude and durability still favoured SRGs. Li et al. (36) found that subacute patients using a force-feedback glove gained 6.1 points on the FMA-UE compared with 3.2 in the control group, with no increase in muscle tone or spasticity. These motor improvements are likely driven by the gloves’ ability to combine volitional effort with real-time sensory and proprioceptive feedback, encouraging sensorimotor integration and cortical reorganization. Functional near-infrared spectroscopy (fNIRS) and EEG studies have further confirmed that SRGs evoke more intense activation in post-lesional motor and premotor regions than MT, suggesting more substantial neuromodulatory effects (44, 45).

MT produces moderate short-term improvements in FMA-UE scores, particularly in subacute patients, but the gains tend to be smaller and less durable. Yavuzer et al. (8) reported a 5.2-point improvement in FMA-UE following 4 weeks of MT in subacute stroke, with a residual benefit at 6 months. However, more recent trials in chronic stroke populations have shown attenuated effects, often limited to distal function and coordination (44). These results may differ from those of our study due to the unique nature of the training protocol used by Zhuang et al. (44), which involved bimanual cooperative tasks with mirror visual feedback. Furthermore, differences in training intensity and duration between studies could also explain the differing outcomes..

As all participants continued to receive their conventional physical therapy rehabilitation, any improvements seen in 1 of the study groups likely resulted from both the standard treatment and the new intervention. These results (within-group comparisons) are included for reference, but should be considered in light of this point. Conventional physical therapy is reported to play a vital role in the rehabilitation of chronic stroke patients, promoting improvements in mobility, muscle strength, and functional independence. However, the rate of recovery may slow during the chronic phase (46).

### Clinical implications

When resources allow, integrating SRGs with standard therapy seems to provide superior, long-lasting improvements in dexterity, strength, and overall upper-limb motor function compared with MT. Mirror therapy remains a low-cost option, particularly soon after a stroke or where robotics is not available, but its benefits may need additional strategies to prevent decay. These outcome-specific insights can help clinicians tailor adjunct therapies to meet individual patient needs and address service limitations.

While this study provides valuable insights, certain limitations should be acknowledged. Although the sample size was sufficient for analysis, a larger sample would enhance the generalizability of the findings to a broader population of post-stroke patients. Additionally, adherence to the prescribed SRGs and MT protocols may have varied among participants, potentially influencing the overall effectiveness of the interventions and long-term outcomes. Furthermore, this study did not include neuroimaging data to assess the neural mechanisms underlying the observed improvements in motor function. Incorporating neuroimaging techniques, such as functional MRI (fMRI), would provide valuable insights into the neuroplastic changes associated with SRGs and MT interventions, helping to elucidate the neural correlates of motor recovery.

In conclusion, the results of this study reinforce the advantages of SRGs over MT in post-stroke hand rehabilitation. SRGs provide targeted, repetitive, and task-specific training that enhances motor recovery more effectively than MT. Notably, the long-term follow-up findings revealed that improvements achieved with SRGs were maintained even after discontinuation of the device, whereas the benefits of MT diminished over time. As research in neurorehabilitation advances, integrating robotic technology into rehabilitation programmes may further improve outcomes for stroke patients, offering a more personalized and effective approach to motor recovery.
